# Contrasting effects of linaclotide and lubiprostone on restitution of epithelial cell barrier properties and cellular homeostasis after exposure to cell stressors

**DOI:** 10.1186/1471-2210-12-3

**Published:** 2012-05-03

**Authors:** John Cuppoletti, Anthony T Blikslager, Jayati Chakrabarti, Prashant K Nighot, Danuta H Malinowska

**Affiliations:** 1 Department of Molecular & Cellular Physiology, University of Cincinnati College of Medicine, 231 Albert Sabin Way, Cincinnati, OH, 45267-0576, USA; 2 Department of Clinical Sciences, College of Veterinary Medicine, North Carolina State University, 4700 Hillsborough Street, Raleigh, NC, 27606, USA

**Keywords:** Linaclotide, Lubiprostone, ClC-2, Epithelia, Barrier function, Restitution, Constipation, CIC, IBS-C

## Abstract

**Background:**

Linaclotide has been proposed as a treatment for the same gastrointestinal indications for which lubiprostone has been approved, chronic idiopathic constipation and irritable bowel syndrome with constipation. Stressors damage the epithelial cell barrier and cellular homeostasis leading to loss of these functions. Effects of active linaclotide on repair of barrier and cell function in pig jejunum after ischemia and in T84 cells after treatment with proinflammatory cytokines, interferon-γ and tumor necrosis factor-α were examined. Comparison with effects of lubiprostone, known to promote repair of barrier function was carried out.

**Results:**

In ischemia-damaged pig jejunum, using measurements of transepithelial resistance, ^3^H-mannitol fluxes, short-circuit current (Cl^−^ secretion) and occludin localization, active linaclotide failed to effectively promote repair of the epithelial barrier or recovery of short-circuit current, whereas lubiprostone promoted barrier repair and increased short-circuit current. In control pig jejunum, 1 μM linaclotide and 1 μM lubiprostone both caused similar increases in short-circuit current (Cl^−^ secretion). In T84 cells, using measurements of transepithelial resistance, fluxes of fluorescent macromolecules, occludin and mitochondrial membrane potential, active linaclotide was virtually ineffective against damage caused by interferon-γ and tumor necrosis factor-α, while lubiprostone protected or promoted repair of epithelial barrier and cell function. Barrier protection/repair by lubiprostone was inhibited by methadone, a ClC-2 inhibitor. Linaclotide, but not lubiprostone increased [cGMP]_i_ as expected and [Ca^2+^]_i_ and linaclotide depolarized while lubiprostone hyperpolarized the T84 plasma membrane potential suggesting that lubiprostone may lead to greater cellular stability compared to linaclotide. In T84 cells, as found with linaclotide but not with lubiprostone, transepithelial resistance was slightly but significantly decreased by guanylin, STa and 8-bromo cGMP and fluorescent dextran fluxes were increased by guanylin. However the physiological implications of these small but statistically significant changes remain unclear.

**Conclusions:**

Considering the physiological importance of epithelial barrier function and cell integrity and the known impact of stressors, the finding that lubiprostone, but not active linaclotide, exhibits the additional distinct property of effective protection or repair of the epithelial barrier and cell function after stress suggests potential clinical importance for patients with impaired or compromised barrier function such as might occur in IBS.

## Background

Linaclotide is a new drug currently under review by the FDA and EMA (European Medicines Agency) for treatment of chronic idiopathic constipation (CIC) and irritable bowel syndrome with constipation (IBS-C) [1-6]. It is a 14 amino acid peptide that gets cleaved *in vivo*, to a 13 amino acid peptide (CCEYCCNPACTGC) by carboxypeptidase action to form MM-419447, the active metabolite [2]. Linaclotide is a homologue of the heat-stable enterotoxin STa, which is in a class of toxins significantly contributing to global endemic diarrhea induced by pathogenic bacteria [5]. Like STa, guanylin and uroguanylin, linaclotide activates guanylate cyclase-C (GC-C) leading to increased [cGMP]_i_[7-9]. In rodent animal models, linaclotide stimulated intestinal fluid secretion and transit time and reduced pain [8-10]. In clinical trials [1-6,11], linaclotide was found to improve bowel symptoms, transit time and abdominal discomfort or pain in patients with IBS-C and CIC. These trials suggest that linaclotide appears to be a safe and effective treatment. However, long term exposure data outside of selected clinical trial populations is not currently available.

Lubiprostone, an analogue of endogenous prostones (functional fatty acids physiologically generated in the human body) is FDA- approved and used for treatment of CIC and IBS-C [12-16]. Lubiprostone activates ClC-2 Cl^-^ channels in the apical membrane of epithelial cells [17,18] thereby increasing intestinal salt and water secretion, promoting bowel movements [19,20] and significantly improving symptoms associated with CIC and IBS-C [12-16]. Lubiprostone also ameliorates abdominal discomfort and pain [13,14] without influencing visceral pain thresholds in patients with IBS-C [21]. In porcine intestine models lubiprostone exhibits an additional distinct property wherein it promotes repair of barrier properties which are disrupted by ischemia, an effect shown to be associated with ClC-2 channel activation [22-24]. These findings indicate that ClC-2 Cl^-^ channels are important in maintenance of the intestinal barrier. The mechanism involved is complex and not fully understood. Repair is mediated in part by restoration of occludin levels and appears to coincide with the movement of occludin back into the apical aspects of tight junctions where ClC-2 is also localized [23]. Most recently it has been shown that ClC-2 modulates tight junction barrier function through intracellular trafficking of occludin [25]. It is additionally of interest that recently it has been found that methadone inhibits lubiprostone-stimulated recombinant ClC-2 Cl^−^ currents and lubiprostone-stimulated Cl^−^ secretion in T84 cells, but has no effect on recombinant CFTR Cl^−^ currents [26].

In contrast, nothing is known about linaclotide effects on epithelial barrier repair after injury or stress. Maintenance of epithelial barrier function and recovery from stressors is important for maintaining normal physiological function of the intestinal tract. Drugs that have reparative properties on barrier function would be helpful in diseased states, where altered intestinal permeability may occur as has been suggested for IBS [27-29], but has seemingly not been investigated in CIC. IBS is characterized by chronic abdominal pain or discomfort with altered bowel function. The pathophysiology of IBS is complex and includes many factors such as visceral hypersensitivity, mucosal immune alterations, psychosocial factors as well as possibly altered intestinal permeability, which however remains controversial [27-32]. In one recent study permeability measurements were carried out in IBS patients and the authors concluded that IBS symptoms were associated with a subtle intestinal permeability increase [27]. Using colonic biopsies of IBS patients (IBS-C, −D, −A) and a fluorescent marker, paracellular permeability was significantly increased in biopsies from IBS patients compared to controls [29]. In addition in this study, Caco-2 cells treated with supernatants from such IBS-patient-derived colonic biopsies showed a significant fall in transepithelial resistance (TER) and lower expression of tight junction protein ZO-1 mRNA compared to healthy individuals [29]. In contrast in another study [30], intestinal permeability was found to be no different in IBS patients compared to healthy controls. However in the same study, NSAIDS compromised intestinal permeability in IBS patients to a greater extent than in healthy subjects, suggesting that IBS is likely associated with altered intestinal barrier responses to noxious agents [30]. More recently, two studies showed that expression and subcellular distribution of the tight junction proteins, ZO-1, occludin and claudin-1 were found to be altered in IBS-C and IBS-D [31] and that paracellular permeability was significantly higher in cecal biopsies from IBS patients compared to controls, with similar increases in all IBS subtypes (−C, −D and −M) [32]. Thus, measuring epithelial barrier properties and epithelial barrier reactions to stressors may reveal additional alterations present in diseased states and studying the effects of compounds used to treat gastrointestinal disorders on such processes may be of considerable clinical importance.

T84 cells grown to confluence develop a high transepithelial resistance (TER) of 1–2 kΩ/cm^2^ and have been used for studies of epithelial barrier function including damage as occurs with the pro-inflammatory cytokines, interferon-γ (IFN-γ) and tumor necrosis factor-α (TNF-α). These cytokines reduce TER in part through reduction of occludin levels and increase passage of high molecular weight molecules eg the endotoxin LPS which may be involved in pathology of celiac sprue enteropathy and inflammatory bowel disease (ulcerative colitis and Crohn’s disease) [33-37].

Using the pig intestine and T84 epithelial cell models, the aim of the present study was to investigate the effects of active linaclotide on barrier function and cellular changes induced by stressors such as ischemia in pig jejunum and IFN-γ and TNF-α, proinflammatory cytokines in T84 cells. Effects of linaclotide were compared with lubiprostone as control, which is known to repair the intestinal epithelial barrier [22-24]. Our results demonstrate by various measures that after injury or stress, active linaclotide failed to effectively repair or protect the epithelial barrier and cell function, whereas lubiprostone repaired or protected the barrier, cell function and homeostasis.

## Methods

### Materials

The active form of linaclotide, MM-419447, (CCEYCCNPACTGC) was prepared by solid phase synthesis and disulfide bridges at 1–6,2–10 and 5–13 were introduced using a random/thermodynamic strategy (6TRT) by GenScript Corporation (Piscataway, NJ) using the 6TRT procedures described by others [38]. The linear peptide had a molecular weight of 1369.58 Da, and as expected the oxidized peptide had a molecular weight of 1363.58. The oxidized peptide purity was 96.2%. The active form of linaclotide was prepared in water. Mitochondrial dye JC-1. DiBAC_4_(3), indo-1 AM, 3,000 Da FITC-dextran or 70,000 Da rhodamine-dextran, mouse anti-occludin and rabbit anti-occludin were obtained from Invitrogen (Eugene, OR). FCCP, fluorescent *E. Coli* lipopolysaccharide (LPS), TNF-α, PGE_1_, 8-bromo cGMP (8BrcGMP), guanylin and ^3^H-mannitol were from Sigma-Aldrich (St. Louis, MO). IFN-γ was from Cell Signaling Technology (Danvers, MA). Mouse anti-beta actin and rabbit anti-beta actin were from Abcam (Cambridge, MA). Protease inhibitor cocktail was from Roche Applied Science (Indianapolis, IN). Cyclic GMP immunoassay kit was from Assay Designs (Ann Arbor, MI). STa was kindly supplied by Dr. Ralph Gianella (University of Cincinnati College of Medicine). Lubiprostone (AMITIZA™) was obtained from R-Tech Ueno (Sanda, Japan) as 2 mM solutions in DMSO.

### Experimental animal surgeries

All studies were approved by the North Carolina State University Institutional Animal Care and Use Committee. Six to eight-week-old Yorkshire crossbred pigs of either sex were housed individually, and maintained on a commercial pelleted feed. Pigs were fasted for 24 h prior to experimental surgery. General anesthesia was induced with xylazine (1.5 mg/kg, IM), ketamine (11 mg/kg, IM), and 5% isoflurane vaporized in 100% O_2_ and was maintained with 2% isoflurane delivered via an endotracheal tube. Pigs were placed on a heating pad and ventilated with 100% O_2_ using a volume-limited, time-cycled ventilator (Hallowell, Pittsfield, MA). Lactated Ringers solution was administered iv at a maintenance rate of 15 ml/kg/h. The jejunum was approached via a ventral midline incision. Jejunal segments were delineated by ligating the intestine at 10 cm intervals, and subjected to ischemia by occluding the local mesenteric blood supply for 45 min.

### Ussing chamber studies and mucosal-to-serosal fluxes of [^3^H]-mannitol

Following the 45 min ischemic period, tissues were harvested from the pig and the mucosa was stripped from the seromuscular layer in oxygenated (95% O_2_/5% CO_2_) Ringers solution (mM: Na^+^, 154; K^+^, 6.3; Cl^−^, 137; HCO_3_^−^, 24; pH 7.4) containing 10 μM indomethacin to prevent endogenous prostaglandin production during the stripping procedure. Tissues were then mounted in 3.14 cm^2^ aperture Ussing chambers, as described in previous studies [22-24]. For Ussing chamber experiments, tissues from one pig were mounted on multiple Ussing chambers and subjected to different *in vitro* treatments such as addition of linaclotide or lubiprostone. Tissues were bathed on the serosal and mucosal sides with 10 ml Ringers solution. The serosal bathing solution contained 10 mM glucose and was osmotically balanced on the mucosal side with 10 mM mannitol. Bathing solutions were oxygenated (95% O_2_/5% CO_2_) and circulated in water-jacketed reservoirs. The spontaneous potential difference (PD) was measured using Ringer-agar bridges connected to calomel electrodes, and the PD was short-circuited through Ag-AgCl electrodes using a voltage clamp that corrected for fluid resistance. TER (Ω/cm^2^) was calculated from the spontaneous PD and short-circuit current (Isc). If the spontaneous PD was between −1.0 and 1.0 mV, tissues were current-clamped at 100 A for 5 s and the PD recorded. Isc and PD were recorded at 15 min intervals over a 180 min experiment.

To assess mucosal permeability after experimental treatments, 0.2 Ci/ml [^3^H]-mannitol (180 Da) was added to the mucosal side of tissues mounted in Ussing chambers. After a 15 min equilibration period, standards were taken from the mucosal side of each chamber and a 60 min flux period was established by taking 0.5 ml samples from the serosal compartment and counting ^3^H radioactivity. Unidirectional [^3^H]-mannitol fluxes from mucosa-to-serosa were determined using standard equations.

### Western analysis of occludin in sucrose density gradient based membrane fractions

Briefly, control or ischemic mucosal samples were homogenized in extraction buffer (50 mM Tris, 25 mM KCl, 5 mM MgCl_2_.6H_2_O, 2 mM EDTA, 40 mM NaF, 4 mM Na_3_VO_4_, pH 7.4) containing 1% Triton X-100 and protease inhibitor cocktail. Homogenized samples were mixed with an equal volume of 80% sucrose in extraction buffer and loaded at the bottom of an ultracentrifuge tube. A discontinuous sucrose gradient was layered on top of the sample by placing 30%, 25%, 20%, and 5% sucrose and the sample was then subjected to ultracentrifugation (250,000 x g, 18 h at 4°C). Ten fractions were removed from the top of each tube and fractions 3 to 10 (fractions 3 to 5 detergent insoluble and fractions 6–10 detergent soluble fraction lanes on blot), as well as whole tissue lysate (normalized amount based on protein content) were used for western blotting for occludin (rabbit anti-occludin antibody, 1:250 dilution). The blots were blocked in 5% milk-TBST for 2 h at room temperature, followed by overnight incubation with primary antibody at 4°C. Secondary antibody was HRP conjugated-goat anti rabbit, and the signals were detected using chemiluminescence. Western analysis was carried out in 3 experiments.

### T84 epithelial cell cultures, TER and experimental procedures

T84 cells (ATCC) were grown in DMEM/Ham’s F-12 medium with 6% heat inactivated FBS, 15 mM HEPES, 14.3 mM NaHCO3, 100 U/ml penicillin, and 100 μg/ml streptomycin sulfate, to confluence on 1.2 cm^2^ filters (Corning Transwell 0.4 μm pore size). TER was measured using an EVOM (Epithelial Volt-Ohm Meter, World Precision Instruments). T84 cells were treated mostly for 3–4 days (if different, it is specified in each legend) with 200 nM of active linaclotide or 100 nM lubiprostone in the absence or presence of 100 ng/ml IFN-γ or 50 ng/ml TNF-α. Control cultures were treated with vehicle only. Active linaclotide was dissolved in water and the vehicle control for lubiprostone was 0.1% DMSO. They are labeled in the figures as control and DMSO respectively.

### Flux of fluorescent dextrans and LPS

T84 cells grown to confluence were treated with compounds/vehicle for 3 days and then incubated with 0.1 mg/ml 3,000 Da FITC-dextran, 0.1 mg/ml 70,000 Da rhodamine-dextran or 0.2 mg/ml FITC labeled E. coli 0111:B4 LPS added to the apical surface for 24 h. FITC (494 nm ex/518 nm em) or rhodamine (570 nm ex/590 nm em) fluorescence of the media bathing the basolateral surface was then measured. For FITC-LPS 530 nm em was used.

### Occludin/actin ratios

T84 cells (10^6^ cells/well) were grown in 12 well Coaster clear bottom plates 48 h prior to the drug treatment. The cells were incubated with or without 200nM active linaclotide, 100 nM lubiprostone, or vehicle for 3 days. Media were changed each day, and fresh active linaclotide, lubiprostone and DMSO were added each day. The cells were washed with cold PBS and lysed with cell extraction buffer containing 1 mM PMSF. T84 cell lysates were added to mouse anti-occludin or mouse anti-β actin coated 96-well clear bottom plates, fixed and stained with rabbit anti-occludin (InVitrogen) or rabbit anti-β actin antibodies (Abcam), followed by streptavidin-HRP labeled secondary antibodies. TMB was used as substrate. HRP-substrate was quantified at 450 nm using a plate reader. The ratio of occludin/actin for each experimental point was calculated. In experiments with 100 ng/ml IFN-γ it was added to the cells in the presence or absence of 200nM active linaclotide, 100 nM lubiprostone, or vehicle for 3 days.

### [Ca^2+^]_i_, plasma membrane potential, [cGMP]_i_ and mitochondrial membrane potential

[Ca^2+^_i_, and plasma membrane potential were measured as previously described [39] using indo-1 AM and the fluorescent membrane potential-sensitive dye, DiBAC_4_(3) respectively before and after addition of compounds at different concentrations. For [cGMP]_i_ T84 cells (10^5^ cells/well) were grown in 96 well plates for 48 h. Compounds/vehicle were added for 2 h and the cells were lysed with 0.1 M HCl-1% Triton X-100 and centrifuged at 600 x g for 5 min. [cGMP]_i_ in T84 cell lysate was measured using a colorimetric cyclic GMP immunoassay kit from Assay Designs (Ann Arbor, MI) following the manufacturer’s instructions.

To measure the mitochondrial membrane potential, T84 cells (10^5^ cells/well) were grown in 96 well Coaster black clear bottom plates for 48 h. The cells were incubated with and without 200 nM active linaclotide, 100 nM lubiprostone and vehicle for 3 days. Media were changed each day, and fresh active linaclotide, lubiprostone and DMSO were added each day. The cells were then washed with HBSS and incubated with 12 μM mitochondrial dye JC-1 in HBSS for 30 min. The plate was read at 490 nm ex/527 nm em and 490 nm ex/590 nm em individually after each experimental condition. 250 nM FCCP was added at the end of each experiment and incubated for 1 h. The membrane potential ratio was 590 nm fluorescence/527 nm fluorescence. The value obtained after FCCP treatment was assigned a value of 0 mV and each individual FCCP ratio was subtracted from each membrane potential ratio value. The control fluorescence ratio was then assigned a value of +224 mV and the mitochondrial membrane potential [μ_H_(mV)] for each experimental point was calculated. In experiments with IFN-γ (100 ng/ml) it was added to the cells in the presence or absence of 200 nM active linaclotide, 100 nM lubiprostone or vehicle for 3 days.

### Statistical analysis

Statistical analysis was carried out using Student’s t-tests for comparison of two unpaired groups. In all cases, the data was normally distributed with equal variance, allowing the use of statistical tests for parametric data. The level of significance was set at *p* < 0.05.

## Results

### Effects of active linaclotide and lubiprostone on (A) TER; (B) paracellular flux of ^3^H-mannitol; (C) western analysis of occludin and (D) I_sc_ in control and ischemic pig intestine

**Figure 1:**
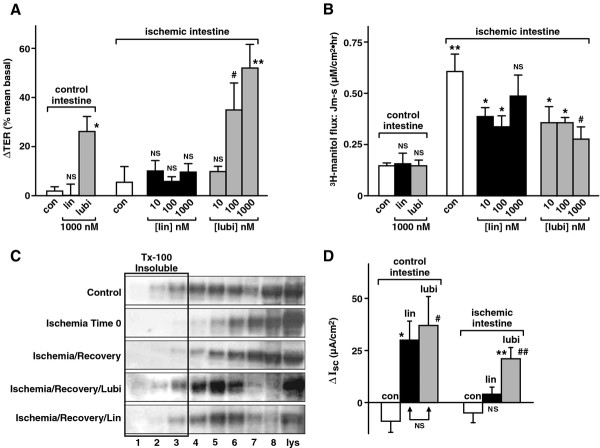
**Effects of active linaclotide and lubiprostone on (A) change in transepithelial electrical resistance (ΔTER), (B) paracellular flux (Jm-s) of**^**3**^**H-mannitol, (C) Western analysis of occludin and (D) Δ Isc in normal and ischemic pig jejunum.** In (**A**) and (**B**) data is plotted as mean ± S.E. (**A**) ΔTER: n = 5; *p < 0.01, **p < 0.005, #*p* < 0.05 and NS, not significant all wrt control. (**B**) ^3^H-mannitol Jm-s: n = 3; *p < 0.05, #*p* < 0.02 and NS, not significant wrt ischemic control; **p < 0.02 ischemia control vs non-ischemic control. (**C**) Western analysis: example of n = 3 experiments. Lanes 1–3, Tx-100 insoluble fractions; lanes 4–8, detergent-soluble fractions; last lane, whole lysate (lys). (**D**) Δ Isc: n = 4; control intestine: *p < 0.025, # *p* < 0.005 wrt control and Δlin vs Δlubi are NS, not significant; ischemic intestine: ** *p* < 0.025 wrt lin, ## *p* < 0.005 wrt ischemic control, NS, not significant wrt ischemic control.

Repairing barrier properties has been shown to be an essential and primary step in recovery after injury and lubiprostone has been shown to repair barrier properties of intestinal epithelia [22-24], while nothing is known regarding linaclotide. Therefore the effects of active linaclotide on barrier properties, occludin and Cl^−^ secretion were examined in control and ischemic pig jejunal mucosa and compared to effects of lubiprostone. The results are shown in Figure 1. In control intestine, 1000 nM (1 μM) active linaclotide had no significant effect on TER, while equimolar lubiprostone (1000 nM or 1 μM) caused a significant increase in TER (from ΔTER = 2.1±1.8 to 26.3 ± 6.2% mean basal (n = 5), p < 0.01) (Figure 1A) and neither compound had any effect on ^3^H-mannitol flux (Figure 1B). In control intestine occludin is present mainly in the detergent-soluble fractions with a small amount in detergent-insoluble fractions (Figure 1C) and both 1 μM linaclotide and 1 μM lubiprostone caused significant and similar increases in Isc as expected (Figure 1D). Ischemia had no effect on TER (Figure 1A), significantly increased ^3^H-mannitol flux from 0.15 ± 0.01 to 0.6 ± 0.1 μM/cm2.h (n = 3, p < 0.02 Figure 1B), decreased the occludin present in all fractions (Figure 1C), suggesting some disruption of tight junctions and had no effect on basal, control Cl^−^ secretion. After ischemia, active linaclotide had no effect on TER (Figure 1A), resulted in a barely significant, dose-independent decrease in ^3^H-mannitol flux (Figure 1B), caused a small increase in occludin (Figure 1C) and had no effect on Isc (Figure 1D). In contrast, after ischemia, lubiprostone resulted in a large, significant increase in TER, decrease in ^3^H-mannitol fluxes, a major return of occludin to detergent-insoluble fractions (Figure 1C, lane 2 and 3) comparable to control and a large, significant increase in Isc. These findings indicate that active linaclotide alone has no effects and does not promote repair of the epithelial barrier after ischemia, while lubiprostone significantly promotes barrier repair, resulting in a tighter and secreting mucosa. Despite the fact that both linaclotide and lubiprostone cause comparable Cl^−^ secretion at equimolar concentrations (Figure 1D), only lubiprostone results in epithelial barrier repair, suggesting that Cl^−^ secretion per se does not lead to repair, whereas functional ClC-2 plays a critical role. To further investigate active linaclotide effects compared to lubiprostone, experiments were conducted on T84 epithelial cells, a system that allows measurement of additional barrier and homeostatic mechanisms in controlled conditions using other pathophysiological stressors.

### Effects of active linaclotide and lubiprostone on IFN-γ- and TNF-A-induced damage to T84 epithelial cell barrier function and mitochondrial membrane potential

**Figure 2:**
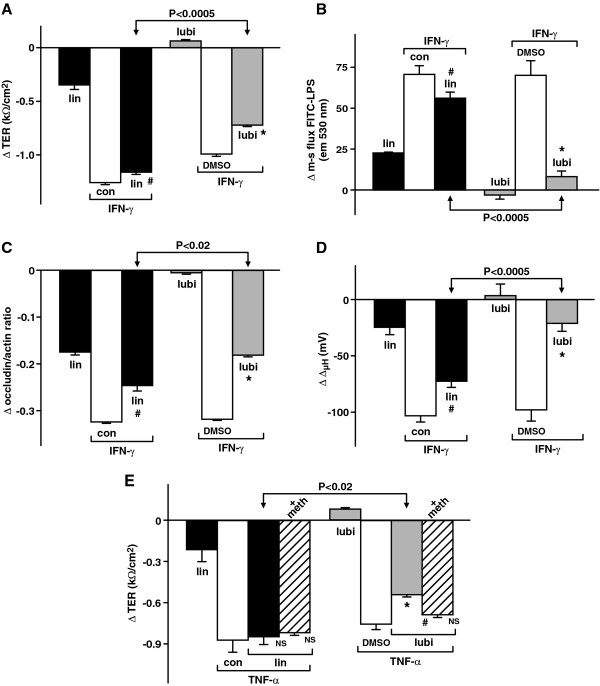
**Effects of active linaclotide and lubiprostone on IFN-γ- induced changes of T84 epithelial cell barrier function and homeostasis: (A) ΔTER; (B) ΔFITC-LPS flux; (C) Δoccludin/actin ratio and (D) ΔΔmitochondrial membrane potential; and (E) Effect of active linaclotide and lubiprostone on TNF-α-induced changes of T84 epithelial cell barrier function measured as ΔTER and effect of methadone.** 200 nM active linaclotide, 100 nM lubiprostone, 100 ng/ml IFN-γ and 50 ng/ml TNF-α were used. Data is plotted as mean ± S.E. (**A**) ΔTER: n = 3, #*p* < 0.02 wrt control, *p < 0.001 wrt DMSO, *p* < 0.0005 for IFN-γ/lin vs IFN-γ/lubi. Basal TERs = 1.56 ± 0.01 kΩ/cm^2^ and 1.26 ± 0.02 for control and DMSO respectively. Δlin vs Δlubi, *p* < 0.0005. (**B**) ΔFITC-LPS flux: n = 3, #*p* < 0.05 wrt control, *p < 0.005 wrt DMSO, *p* < 0.0005 for IFN-γ/lin vs IFN-γ/lubi. Basal LPS fluxes = 361.1 ± 3.4 and 378.1 ± 3.6 em units @ 530 nm for control and DMSO respectively. Δlin vs Δlubi, *p* < 0.01. (**C**) Δoccludin/actin ratio: n = 3, #*p* < 0.02 wrt control, *p < 0.0005 wrt DMSO, *p* < 0.0005 for IFN-γ/lin vs IFN-γ/lubi. Basal ratios = 0.98 ± 0.01 and 0.98 ± 0.01 for control and DMSO respectively. Δlin vs Δlubi, *p* < 0.0005. (**D**) ΔΔmitochondrial membrane potential: n = 8, #*p* < 0.001 wrt control, *p < 0.0005 wrt DMSO, *p* < 0.0005 for IFN-γ/lin vs IFN-γ/lubi. Basal Δ mito pds = 224.0 ± 0.2 and 211.1 ± 8.8 mV for control and DMSO respectively. Δlin vs Δlubi, *p* < 0.0005. (**E**) ΔTER: 1 μM methadone was used. NS, not significant wrt TNF-α/control, *p < 0.01 wrt TNF-α/DMSO, # *p* < 0.005 wrt TNF-α/lubi and *p* < 0.02 for TNF-α/lin vs TNF-α/lubi. Basal TERs = 1.32 ± 0.06 and 1.19 ± 0.01 kΩ/cm^2^ for control and DMSO respectively. Δlin vs Δlubi, *p* < 0.05.

IFN-γ, a pro-inflammatory cytokine, reduces TER in part through reduction of occludin levels and is thought to be involved in the pathology of celiac sprue enteropathy and inflammatory bowel disease (ulcerative colitis and Crohn’s disease) [33-37]. Similar to IFN-γ, TNF-α also causes loss of barrier function [34,36,37]. Lubiprostone has been shown to promote repair of intestinal barrier properties after ischemia in the pig intestine model [22-24] whereas nothing is known of the effect of linaclotide. Therefore the effects of active linaclotide and lubiprostone on IFN-γ- and TNF-α-induced loss/disruption of T84 epithelial cell barrier and cell function were next examined. TER, mucosal to serosal FITC-LPS flux, occludin/actin ratio and mitochondrial membrane potential were measured. The results shown in Figure 2 are plotted as changes compared to vehicle controls (basal levels are given in the legend). IFN-γ (100 ng/ml) significantly reduced TER (Figure 2A, p < 0.0005), increased mucosal to serosal FITC-LPS flux (Figure 2B, p < 0.0005), decreased the occludin/actin ratio (Figure 2C, p < 0.0005) and decreased the mitochondrial membrane potential (Figure 2D, p < 0.0005). Active linaclotide had small IFN-γ-counteracting effects (ca. 5.7%, 20%, 23% and 30%, Figures 2A, B,C and D respectively), while lubiprostone had greater effects (ca. 27.3%, 89%, 43.8% and 77.8%, Figure 2A, B, C and D respectively), partially or totally preventing/repairing the effects of IFN-γ. The significance of the Δlinaclotide versus Δlubiprostone in the presence of IFN-γ for Figure 2A2B2C &2D were p < 0.0005, p < 0.0005, p < 0.02 and p < 0.0005 respectively. Guanylin (200 nM), STa (50 nM) and 8Br-cGMP (1 mM) had effects similar to active linaclotide (data not shown). Active linaclotide was relatively ineffective, while lubiprostone was significantly effective in protecting from or repairing the detrimental effects of IFN-γ on T84 epithelial cell barrier function and cell homeostasis. Thus, after IFN-γ lubiprostone, but not linaclotide, protected or repaired barrier and cell function.

As shown in Figure 2E, TNF-α (50 ng/ml) also significantly reduced T84 cell TER (p < 0.0005). Active linaclotide had no effect on these changes, but lubiprostone partially protected/repaired barrier function after TNF-α. Δlin wrt Δlubi were significantly different (p < 0.02). Therefore after TNF-α’s detrimental effects, lubiprostone, but not linaclotide, significantly protected or repaired epithelial barrier function. Methadone has been shown recently to inhibit recombinant ClC-2, but not recombinant CFTR Cl^−^ currents and to inhibit lubiprostone-stimulated Isc in T84 cells [26]. Therefore the effect of 1 μM methadone on lubiprostone’s protective/reparative properties after TNF-α, as measured by TER, was also investigated and compared with linaclotide’s effects. The results are also shown in Figure 2E. Methadone inhibited completely lubiprostone’s protective or reparative effects on TER after TNF-α and had no effect on linaclotide’s lack of barrier proetection or repair. These findings confirm that lubiprostone’s protective or reparative effects in T84 cells are mediated by functional ClC-2.

Stressors (inflammatory cytokines) had large effects on epithelial barrier function and the mitochondrial membrane potential. However even in the absence of stressors or injury, there were also small, but statistically significant negative changes evident with linaclotide alone. In contrast lubiprostone alone had no negative effects on the measured parameters. The physiological significance of the small but statistically significant differences between effects of linaclotide and lubiprostone alone remain unclear.

### Effects of active linaclotide and lubiprostone on T84 (a) [cGMP]I, (B) [Ca2+]I and (C) plasma membrane potential

**Figure 3:**
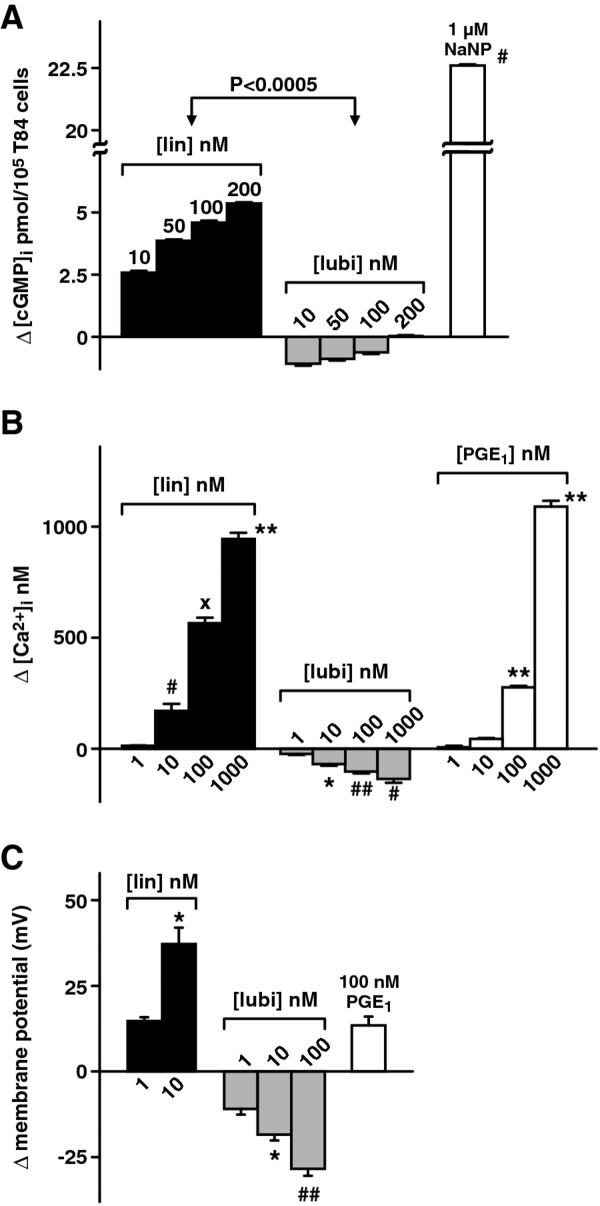
**Comparison of effects of active linaclotide and lubiprostone on T84 cell homeostasis: (A) Δ[cGMP]**_**i**_**; (B) Δ[Ca**^**2+**^**]**_**i**_**; (C) Δplasma membrane potential.** 200 nM active linaclotide and 100 nM lubiprostone were used. Data is plotted as mean ± S.E. (**A**) Δ[cGMP]_i_: n = 5, *p* < 0.0005 lin vs lubi. 1 μM NaNP was the positive control. Basal [cGMP]_i_s =1.7 ± 0.001, 3.1 ± 0.01 and 23.9 ± 0.002 pmol/10^5^T84 cells for lin, lubi and PGE_1_ respectively. (**B**) Δ[Ca^2+^]_i_: n = 3, *p < 0.01, **p < 0.0005, #*p* < 0.02, ##*p* < 0.0025, x*p* < 0.001 all wrt 1 nM drug. Basal [Ca^2+^]_i_s = 175.5 ± 7.1, 117.6 ± 6.4 and 80.8 ± 2.4 nM for lin, lubi and PGE_1_ respectively. (**C**) Δplasma membrane potential: n = 5, ##*p* < 0.001, *p < 0.02 all wrt 1 nM drug. PGE_1_ was used as positive control.

Increases in [cGMP]_i_ and [Ca^2+^_i_ have been shown with linaclotide and guanylin and changes in occludin occur with breakdown of barrier function [8,33,35,36,40]. Therefore active linaclotide effects on [cGMP]_i_, [Ca^2+^_i_ and plasma membrane potential were investigated and compared with effects of lubiprostone. The results are shown in Figure 3 as changes that occurred compared to vehicle controls (basal levels are given in the legend). Active linaclotide significantly increased [cGMP]_i_ while lubiprostone significantly decreased [cGMP]_i_ (Figure 3A, p < 0.0005). Active linaclotide and PGE1 significantly increased [Ca^2+^_i_, while lubiprostone significantly decreased [Ca^2+^_i_ (Figure 3B, p values are given in the figure legend). Figure 3C shows that active linaclotide significantly depolarized the plasma membrane potential, while lubiprostone significantly hyperpolarized the plasma membrane potential (p values are given in the figure legend). Thus active linaclotide, but not lubiprostone, resulted in increased [cGMP]_i_ as expected and increased [Ca^2+^_i_. Effects on the plasma membrane potential suggest that lubiprostone, but not linaclotide, leads to cell stabilization, that helps maintain cellular homeostasis.

### Effects of active linaclotide compared to guanylin, STa. and 8BrcGMP on T84 cells

**Figure 4:**
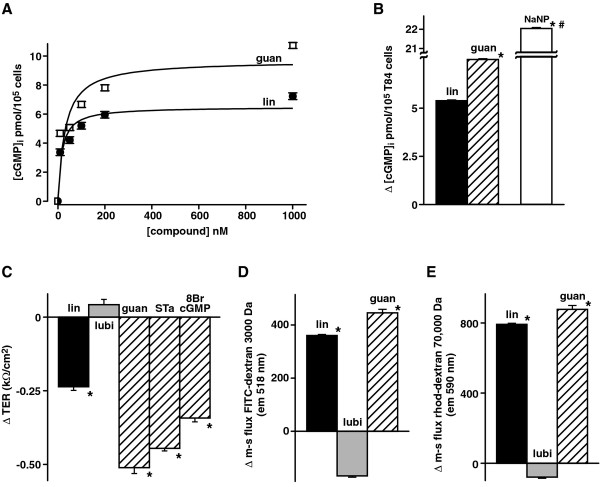
**Effects of active linaclotide and guanylin on T84 cells: (A) Δ[cGMP]**_**i**_**dose response; (B) effect of 1 μM Na nitroprusside on Δ[cGMP]**_**i**_**; and Effects of active linaclotide, guanylin, STa and 8BrcGMP on (C) ΔTER; (D) Δ3,000 Da FITC-dextran flux; (E) Δ70,000 Da rhodamine-dextran flux.** 200 nM active linaclotide, 100 nM lubiprostone, 200 nM guanylin, 50 nM STa and 1 mM 8BrcGMP were used. Data is plotted as mean ± S.E. (**A**) Δ[cGMP]_i_ dose response: EC_50_ lin = 15.9 ± 7.4 nM (n = 6); EC_50_ guan = 29.3 ± 18.4 nM (n = 6), Vmax lin = 6.5 ± 0.6 pmol/10^5^T84 cells and Vmax guan = 9.7 ± 1.3 pmol/10^5^ T84 cells. (**B**) Effect of 1 μM Na nitroprusside on Δ[cGMP]_i_: n = 5, *p < 0.0005 wrt lin, #*p* < 0.0005 wrt guan. Basal [cGMP]_i_ = 1.55 ± 0.002 pmol/10^5^ T84 cells. (**C**) ΔTER: n = 3, *p < 0.0005 wrt lubi. For lubi, lin and guan, basal DMSO TER =1.21 ± 0.02 kΩ/cm^2^ and basal control = 1.35 ± 0.04 kΩ/cm^2^; for STa and 8BrcGMP, basal control = 1.29 ± 0.02 kΩ/cm^2^. (**D**) Δ3,000 Da FITC-dextran flux: n = 3, *p < 0.0005 wrt lubi. Basal control flux = 620.9 ± 2.2 and basal DMSO flux = 902.9 ± 19.9 em units @ 518 nm. (**E**) Δ70,000 Da rhodamine-dextran flux: n = 3, *p < 0.0005 wrt lubi. Basal control flux = 1759.4 ± 21.8 and basal DMSO flux = 2240.7 ± 13.1 em units @ 590 nm.

Linaclotide has major significant structural homologies with guanylin, uroguanylin and especially heat stable enterotoxin, STa [5,8,41], all of which (including linaclotide) activate guanylate cyclase, GC-C resulting in increased [cGMP]_i_. Therefore effects of active linaclotide on T84 cells were compared with those of guanylin, STa and 8BrcGMP. Both active linaclotide and guanylin significantly increased [cGMP]_i_ in a dose-dependent manner with EC_50_ values of 15.9 ± 7.4 and 29.3 ± 18.4 nM (n = 6) respectively as shown in Figure 4A &4B. Figure 4C shows that like 200 nM linaclotide, 200 nM guanylin, 50 nM STa and 1 mM 8BrcGMP all reduced T84 epithelial cell TER significantly (p < 0.0005) as compared with 100 nM lubiprostone which caused a slight increase in TER. Figure 4D &4E show that like 200 nM linaclotide, 200 nM guanylin resulted in significantly (p < 0.0005) increased mucosal to serosal fluxes of 3,000 Da FITC-dextran (Figure 4D) and 70,000 Da rhodamine-dextran (Figure 4D) compared to lubiprostone which resulted in decreases of these fluxes. These effects on epithelial barrier function were small compared to effects of stressors (see Figure 2) and their physiological relevance or significance remains unclear. Nevertheless, these findings confirm that active linaclotide and well established GC-C activators such as guanylin and STa and the membrane permeant 8BrcGMP have similar effects on barrier function of T84 cells.

## Discussion

Linaclotide is being developed for treatment of CIC and IBS-C [1-6]. Like lubiprostone, linaclotide has been reported to increase chloride and water secretion and exert antinociceptive effects in rats [8-10]. In clinical trials, like lubiprostone, linaclotide improved bowel symptoms, transit time and abdominal discomfort or pain in patients with ClC and IBS-C [1-6,11-16,19,20]. Linaclotide appears to be safe and effective and is currently under review by the FDA and EMA, while lubiprostone is FDA-approved for use in ClC and IBS-C. Unlike lubiprostone that activates the ClC-2 Cl^−^ channel [17,18], linaclotide acts through stimulation of guanylate cyclase C resulting in increased [cGMP]_i_[7-9]. Comparison of these two drugs is of particular interest since they have different cellular mechanisms of action. An additional distinct property of lubiprostone is its ability to repair/protect epithelial barrier function after injury or exposure to stressors [22-24]. ClC-2 has been shown to be important for maintenance and repair of barrier function after injury, which also coincided with occludin movement back into apical aspects of the epithelial tight junctions where ClC-2 is also localized [23]. In fact it has been shown that ClC-2 modulates tight junction barrier function via intracellular trafficking of occludin [25]. Whether linaclotide also exhibits this barrier reparative/protective property after injury or exposure to stressors has not been previously studied.

Active linaclotide, the 13 amino acid peptide produced by hydrolysis of the pro-drug linaclotide, used in the present study, increased [cGMP]_i_ in T84 cells by activating GC-C, demonstrating that the peptide was folded properly. Positive controls included guanylin which also activates GC-C, and NaNP, which activates the soluble form of guanylate cyclase, yielding much higher levels of [cGMP]_i_ in T84 cells than linaclotide.

Lubiprostone, but not active linaclotide was effective at promoting repair of the pig jejunal intestinal mucosa after acute ischemic injury, as measured by TER changes, ^3^H-mannitol fluxes and cellular occludin localization. Although Cl^−^ secretion as measured by short-circuit current was similar in control intestine stimulated by linaclotide and lubiprostone (both at 1 μM), after ischemia, only lubiprostone, but not linaclotide resulted in Cl^−^ secretion, not significantly different from control intestine. Thus epithelial barrier properties and Cl^−^ secretion were repaired/returned close to normal with lubiprostone, but not linaclotide. Despite the fact that both linaclotide and lubiprostone cause Cl^−^ secretion, only lubiprostone resulted in epithelial barrier repair, suggesting that Cl^−^ secretion *per se* does not lead to repair, whereas functional ClC-2 plays an essential role [22-25].

Experiments were carried out on T84 cells treated with IFN-γ or TNF-α to investigate whether effects of inflammatory cytokines as stressors could also be attenuated by linaclotide or lubiprostone. The lubiprostone concentration chosen for these studies (100 nM) was 5 x EC_50_ for Cl^−^ secretion activation [17]. The concentration of linaclotide was chosen to be twice that of lubiprostone, although the molar concentration ratio of linaclotide/lubiprostone used in treatment of CIC in the clinical setting is 3:1 [1,12]. Similar to effects on pig jejunal mucosal barrier, in T84 cells active linaclotide was not very effective at protecting or repairing barrier properties after injury by either IFN-γ or TNF-α, as measured by ΔTER and Δfluorescent LPS flux. Neither was it very effective at preventing injury-induced mitochondrial potential depolarization or causing return of occludin levels to normal. In contrast lubiprostone was very effective at protecting or repairing barrier and cell function as well as occludin levels after cytokine injury. Lubiprostone effects on the epithelial mucosal barrier were expected since it has been previously shown to promote repair of intestinal mucosa barrier properties after ischemia [22-24]. In addition, the protective/reparative effects of lubiprostone on TNF-α-induced decreased TER were completely inhibited by 1 μM methadone, a ClC-2 inhibitor [26]. Methadone had no effect on linaclotide effects. These findings indicate that lubiprostone’s barrier reparative properties are mediated by ClC-2, also supported by the fact that barrier repair is lacking in ClC-2^−/−^ mice [23].

The importance of the reparative properties of lubiprostone for IBS-C and CIC is unclear, since the role of intestinal permeability remains controversial in IBS-C [27-32] and seemingly has not been investigated in CIC. In addition IBS-C and CIC are symptomatic diseases which do not appear to have clear physiological or biochemical markers that define them. However, in the future these properties may prove to be important for treatment of other intestinal diseases, where increased intestinal permeability or inflammation (which may release inflammatory cytokines) appear to occur [33-37].

In the present study, effects of active linaclotide alone on T84 cell properties and parameters were noted, and they were opposite to those seen with lubiprostone. However although statistically significant, these effects were relatively small compared to stressor effects and they are difficult to relate to the physiology of intestinal mucosal tissue or the intact animal. Active linaclotide increased [cGMP]_i_ as expected [7-9], increased [Ca^2+^_i_ and caused depolarization of both the mitochondrial membrane potential and the plasma membrane potential. One of the consequences of high [cGMP]_i_ (whether from STa activation of GC-C or exogenous 8BrcGMP) includes activation of a cyclic nucleotide gated Ca^2+^ channel causing increased [Ca^2+^_i_[40], which could cause depolarization of both mitochondrial and plasma membrane potentials. As found with linaclotide, guanylin, STa and 8BrcGMP decreased TER in T84 cells and guanylin was to found to increase fluxes of fluorescent dextrans. Therefore linaclotide clearly acts similarly to well established GC-C activators and membrane permeant cGMP. Guanylin, uroguanylin and STa also activate K^+^ channels by a separate mechanism [7]. If linaclotide also has such effects, they may be responsible for the depolarization of the intestinal plasma membrane. However linaclotide effects on K^+^ channels were not studied in the present study. In contrast, lubiprostone had no effect on [cGMP]_i_, significantly decreased [Ca^2+^_i_, had no effect on the mitochondrial membrane potential and caused hyperpolarization of the plasma membrane potential. These cellular effects likely render the cells in a more stabilized state, while linaclotide has the opposite effect. These findings distinguish linaclotide from lubiprostone and the prostones in general, which do not change [cGMP]_i,_ [Ca^2+^_i_ or mitochondrial membrane potential and hyperpolarize rather than depolarize the plasma membrane potential. These effects may contribute not only to cell stability, but also may play a role in lubiprostone’s reparative/protective properties.

Both linaclotide and lubiprostone ameliorate clinical symptoms of CIC and IBS-C. However they have different mechanisms of action and have different cellular effects. Linaclotide does not appear to have the additional distinct epithelial barrier reparative/protective properties of lubiprostone.

## Conclusions

In summary, in this *in vitro* study, active linaclotide did not exhibit the distinct epithelial barrier reparative properties shown by lubiprostone after ischemic injury to the pig intestine or lubiprostone’s protective/reparative properties after inflammatory cytokine exposure of T84 cells. Lubiprostone’s barrier protective/reparative effect after TNF-α in T84 cells was abolished by methadone, a ClC-2 inhibitor, indicating that lubiprostone’s effects are mediated by ClC-2. Linaclotide increased [cGMP]_i_ as expected, but also increased [Ca^2+^]_i_, and caused depolarization of both the mitochondrial and plasma membrane potentials. This was in contrast/opposite to lubiprostone that had no effect on [cGMP]_i_, [Ca^2+^]_i_ and mitochondrial membrane potential and hyperpolarized the plasma membrane potential. As found with linaclotide, guanylin, STa and 8BrcGMP decreased TER in T84 cells and guanylin also increased fluxes of fluorescent dextrans. Therefore linaclotide clearly acts similarly to well established GC-C activators and exogenous membrane permeant cGMP. Stressors can induce pathophysiological changes in barrier function. Considering the physiological importance of epithelial barrier function and cell integrity and the known impact of stressors, lubiprostone, but not active linaclotide, exhibits the additional distinct property of protecting or repairing the epithelial barrier and cell function after stress. This may be beneficial to patients with impaired or compromised epithelial barrier function such as might occur in IBS.

## Abbreviations

IFN-γ: Interferon-γ; TNF-α: Tumor necrosis factor-α; TER: Transepithelial resistance; LPS: Lipopolysaccaride; IBS: Irritable bowel syndrome; ClC: Chronic idiopathic constipation; IBS-C: Irritable bowel syndrome with constipation; STa: Heat-stable enterotoxins; GC-C: Guanylate cyclase-C; JC-1: 5,5′,6,6′-tetrachloro-1,1′,3,3′–tetraethylbenzimidazolyl-carbocyanine chloride; DiBAC4(3): Bis-(1,3-dibutylbarbituric acid)trimethine oxonol; indo-1AM: 1H-Indole-6-carboxylic acid; 2-[4-[bis[2-[(acetyloxy)methoxy]-2-oxoethyl]amino]-3-[2-[2-[bis[2-[(acetyloxy)methoxy]-2-oxoethyl]amino]-5-methylphenoxy]ethoxy]phenyl]-: (acetyloxy)methyl ester; FITC: Fluorescein isothiocyanate; FCCP: Carbonyl cyanide P-(trifluoromethoxy) phenylhydrazone; PGE1: Prostaglandin E1; DMSO: Dimethylsulfoxide; PD: Potential difference; Isc: Short-circuit current; TBST: Tris-buffered saline with Tween 20; HRP: Horseradish peroxide; FBS: Fetal bovine serum; HBSS: Hanks buffered salt solution; PBS: Phosphate buffered saline; PMSF: phenylmethanesulfonyl fluoride; TMB: 3,3′,5,5′-tetramethylbenzidine; 8BrcGMP: 8-bromo cGMP; NaNP: Sodium nitroprusside; lin: Linaclotide; lubi: Lubiprostone.

## Competing interest

John Cuppoletti and Danuta H. Malinowska have financial interests in Sucampo companies, including research support and consulting fees from Sucampo AG, Zug, Switzerland and John Cuppoletti has stock options in Sucampo. Anthony T. Blikslager and Prashant K. Nighot have funding from Sucampo Pharmaceutical Americas and Anthony T. Blikslager is a consultant to Sucampo.

## Authors’ contributions

JC, DHM and ATB designed the research and wrote the paper. JCh and PKN performed the research. All authors read and approved the final manuscript.
